# Rsite2: an efficient computational method to predict the functional sites of noncoding RNAs

**DOI:** 10.1038/srep19016

**Published:** 2016-01-11

**Authors:** Pan Zeng, Qinghua Cui

**Affiliations:** 1 Department of Biomedical Informatics, Centre for Noncoding RNA Medicine, School of Basic Medical Sciences, Peking University, 38 Xueyuan Rd, Beijing, 100191, China

## Abstract

Noncoding RNAs (ncRNAs) represent a big class of important RNA molecules. Given the large number of ncRNAs, identifying their functional sites is becoming one of the most important topics in the post-genomic era, but available computational methods are limited. For the above purpose, we previously presented a tertiary structure based method, Rsite, which first calculates the distance metrics defined in Methods with the tertiary structure of an ncRNA and then identifies the nucleotides located within the extreme points in the distance curve as the functional sites of the given ncRNA. However, the application of Rsite is largely limited because of limited RNA tertiary structures. Here we present a secondary structure based computational method, Rsite2, based on the observation that the secondary structure based nucleotide distance is strongly positively correlated with that derived from tertiary structure. This makes it reasonable to replace tertiary structure with secondary structure, which is much easier to obtain and process. Moreover, we applied Rsite2 to three ncRNAs (tRNA (Lys), Diels-Alder ribozyme, and RNase P) and a list of human mitochondria transcripts. The results show that Rsite2 works well with nearly equivalent accuracy as Rsite but is much more feasible and efficient. Finally, a web-server, the source codes, and the dataset of Rsite2 are available at http://www.cuialb.cn/rsite2.

RNAs, in conventional view, comprise mostly messenger RNAs (mRNAs) that are used by cells merely to convey genetic information, and several typical noncoding RNAs (ncRNAs), including transfer RNA (tRNA) and ribosomal RNA (rRNA), which are directly involved in protein translation. However, as the rapid development of high-throughput RNA-sequencing biotechnologies, a large number of previously un-annotated ncRNAs have been uncovered^1^,^2^, which mainly contain microRNAs (miRNAs) and long noncoding RNAs (lncRNAs)^3^. Recently, accumulating studies have shown that ncRNAs play critical roles in a variety of biological processes and thus are associated with a wide spectrum of diseases^4^,^5^,^6^,^7^. Although they cannot encode proteins, ncRNAs are important regulators inside the complex regulatory networks of homeostasis^8^,^9^. Therefore, ncRNAs are essential to maintain health and are considered to have potential for disease diagnosis and therapy.

Given the large number of ncRNAs, it becomes increasingly urgent to annotate them. For this purpose, computational methods have a significant contribution^10^. In the post-genomic era, one of the most important topics is identifying the functional sites of ncRNAs for better understanding of their functions. However, methods for the above purpose remain limited. The diverse functions of RNAs, especially ncRNAs, are not only conferred by their nucleotide sequences, but also endowed by the secondary, tertiary and even quaternary structures^11^,^12^,^13^. To infer functional sites, several biochemical methods have been developed^14^, such as SHAPE and mutational profiling (SHAPE-MaP)^15^, RNase footprinting^16^, hydroxyl radical footprinting^17^, and short-range RNA-RNA crosslinking^18^. These methods contribute much to the identification of the functional sites of RNAs. However, they are rather laborious, time consuming and not economical. In addition, it is hard to keep up with the fast increasing number of ncRNAs. Moreover, the above approaches only work well on identifying big RNA domains or RNA motifs but perform poorly on identifying functional sites whose lengths range from one nucleotide to several nucleotides. Thus, developing alternative computational methods is increasingly needed.

In contrast, for proteins, one big class of more well-studied biological molecules, there are a number of in silico methods for the identification of their functional sites. Given that many public databases have been created for proteins to store related experimental data, such as protein 3D structures^19^, protein-protein interactions^20^,^21^,^22^, and known protein functional sites^23^,^24^,^25^,^26^, a myriad of computational methods for predicting protein functional sites have been released based on the above datasets^27^. These methods use diverse strategies, e.g. machine learning^28^, pattern recognition^29^, phylogenetic motif identification^30^,^31^,^32^, structure network analysis^33^, text mining^34^, and geometric analysis^35^,^36^,^37^,^38^. However, considering currently limited information about ncRNA functional sites and low sequence conservation of ncRNAs^39^, computational methods like machine learning and phylogenetic motif identification are not suitable so far to predict ncRNA functional sites. As for geometric analysis, it finds that the residues close to the protein centroid^38^ and the residues that resides on the protein surface^35^ could be the functional elements. These residues are normally located within the local extreme points of residue distance curve. We suppose that it could be applicable for ncRNA as well. In a previous study^40^, we have developed a tertiary geometry based computational method, Rsite, to identify ncRNA functional sites and have displayed its reliable accuracy. The major limitation of Rsite is that it works based on ncRNA tertiary structures. Up to now for most of ncRNAs, their experimentally derived tertiary structures are not available. In addition, it is still very hard to identify the tertiary structures of RNAs especially large RNA molecules (>=100nt) using computational prediction^41^,^42^,^43^. The application of Rsite is therefore largely restricted.

In this study, we first revealed a strong correlation between the nucleotide distances derived from the RNA tertiary structures and those derived from the RNA secondary structures. This makes it reasonable to develop secondary structure geometry based computational methods for ncRNA functional site prediction. Compared with ncRNA tertiary structures, secondary structures can be determined and predicted more simply and easily. Based on the above observation, here we developed a secondary structure based computational method, Rsite2, to predict the functional sites of ncRNAs. Finally, we confirmed that Rsite2 has a nearly equivalent accuracy as the tertiary structure based method (Rsite) but is much more feasible and efficient.

## Results

### ncRNA secondary structures and tertiary structures show significantly positive correlation

**Figure 1:**
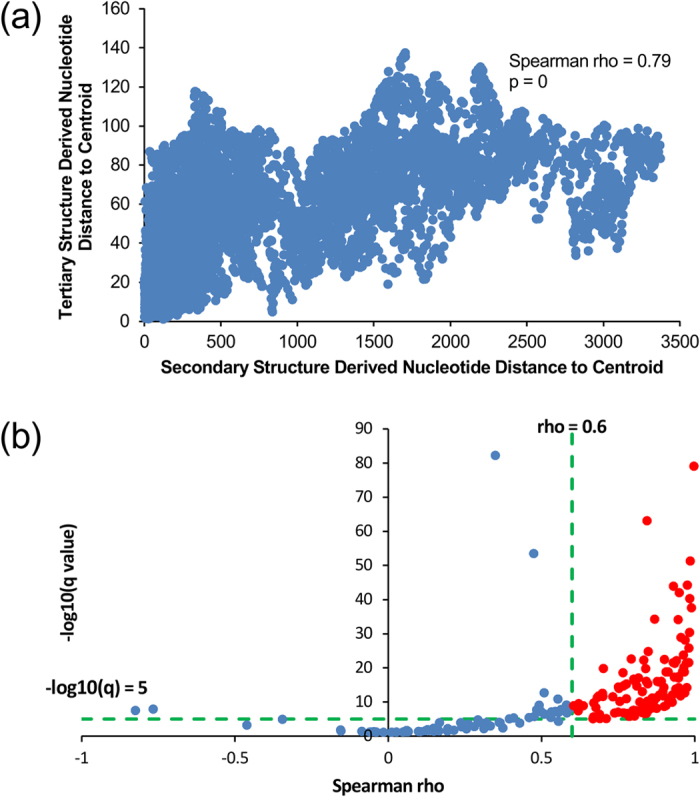
ncRNA secondary structure positively correlates with tertiary structure significantly. (**a**) Nucleotide distances to corresponding centroids derived from the tertiary structures plotted versus those derived from the secondary structures of the 203 ncRNAs (Spearman rho = 0.79, p = 0). (**b**) −log_10_(q) plotted versus Spearman correlation coefficients of the 203 ncRNAs. Data points of ncRNAs that display significantly strong correlations (Spearman rho >0.6, q < 1e-5) are in red.

With a non-redundant list of RNA-containing 3D structures from the Nucleic Acid Database (NDB, http://ndbserver.rutgers.edu/)^44^,^45^ that records in total 1419 PDB IDs of the equivalence class representatives, we obtained 1034 biological assemblies of them from the Protein Data Bank (PDB, http://www.rcsb.org/pdb/home/home.do)^19^. After a screening procedure (see Methods), there left 203 ncRNA molecules for further 2D-3D structure correlation analysis. Almost all of the 203 ncRNAs are shorter than 200nt except a few rRNA. In the case of the Euclidean distances from nucleotides to molecular centroid, the overall Spearman correlation coefficient is 0.79 (p = 0) (Fig. 1a). In addition, for each specific ncRNA molecule, we calculated the q values using the R (v3.2.0) package ‘qvalue’^46^ (v1.99.1). It can be seen that 122 (60%) of the 203 ncRNAs exhibit significantly strong correlations (Spearman rho >0.6, q < 1e-5) (Fig. 1b) and 173 (85%) ncRNA molecules demonstrate significantly positive correlations (q < 0.01) between 2D and 3D structures.

As shown above, it is reasonable to predict ncRNA functional sites on the basis of secondary structure-derived distance metrics. And we further proved the feasibility in the following sections.

### Predicting the functional sites of the tRNA (Lys)

**Figure 2:**
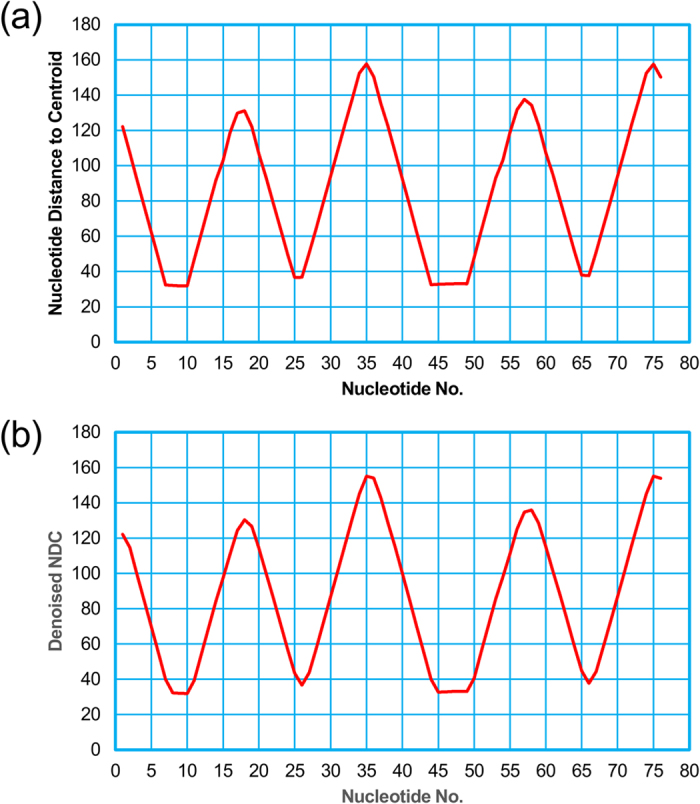
The raw nucleotide distance curve (**a**) and the smoothed nucleotide distance curve (**b**) of the tRNA (Lys).

**Figure 3:**
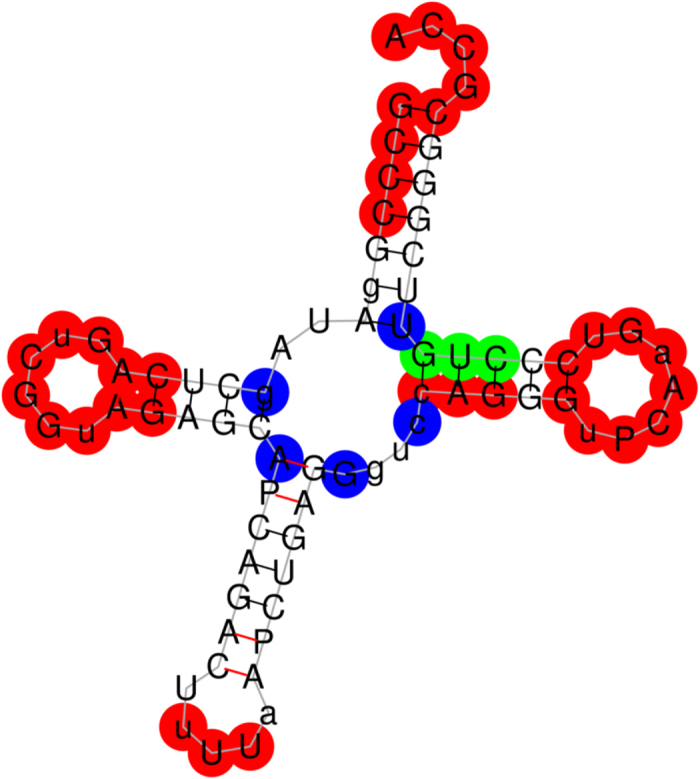
The predicted functional sites of the tRNA (Lys) in a graphical representation. The figure shows the secondary structure of the tRNA (Lys). A predicted functional site is colored red on condition that it hits a known functional site, otherwise blue. And the missed known functional sites are in green color.

Firstly, we downloaded the secondary structure of the tRNA (Lys) in PostScript format from RNA STRAND (http://www.rnasoft.ca/strand/)^47^. We next calculated and plotted the distance curve (Fig. 2). As a result, Rsite2 identified 10 possible functional sites in total. The result shows that only 1 of the 7 known functional sites is missed (Table 1; Fig. 3). As a result, Rsite2 has a sensitivity of 86% (6/7) and a positive predictive value (PPV) of 60% (6/10) for the prediction of the functional sites of tRNA (Lys).

### Predicting the functional sites of the Diels-Alder ribozyme

**Figure 4:**
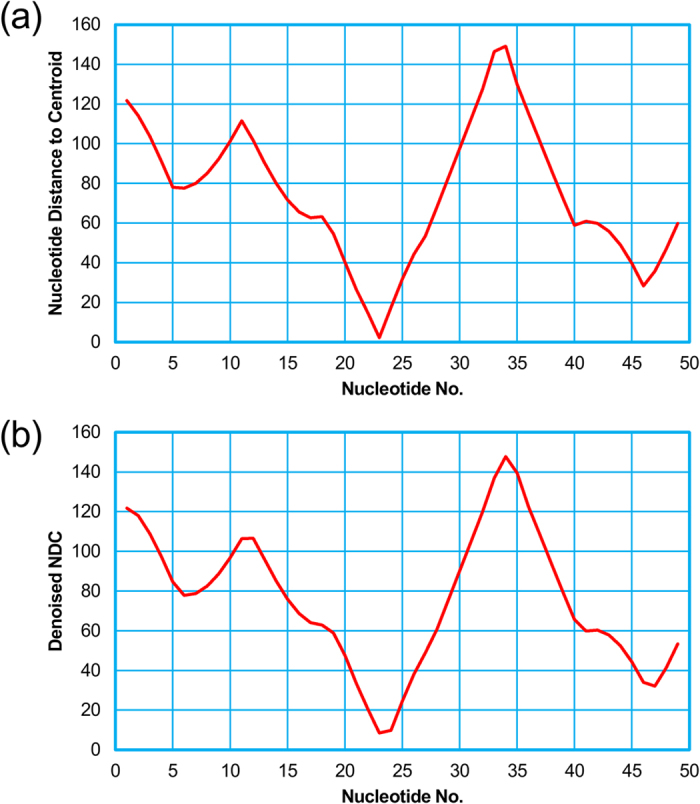
The raw nucleotide distance curve (**a**) and the smoothed nucleotide distance curve (**b**) of the Diels-Alder ribozyme.

**Figure 5:**
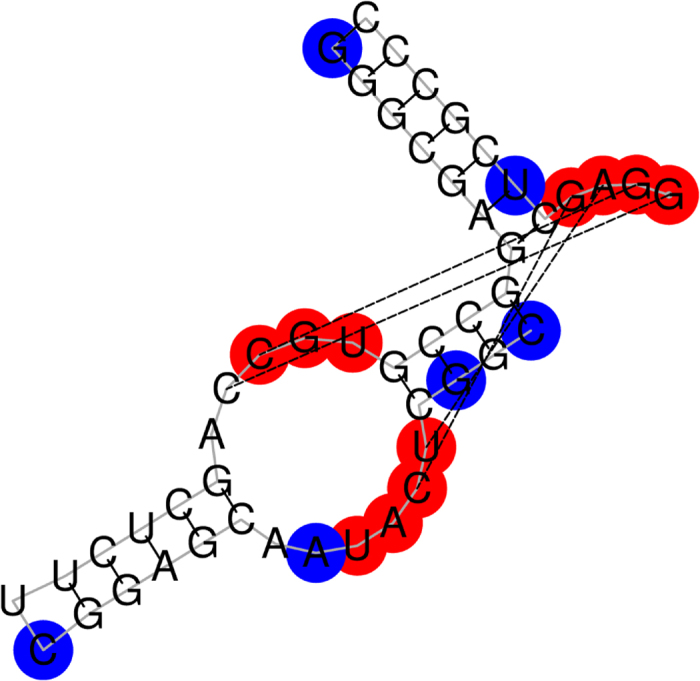
The predicted functional sites of the Diels-Alder ribozyme in a graphical representation. The figure shows the secondary structure of the Diels-Alder ribozyme. A predicted functional site is colored red on condition that it hits a known functional site, otherwise blue. And the missed known functional sites are in green color.

In the same way as the above section, we calculated and plotted the distance curve of the Diels-Alder ribozyme (Fig. 4). As a result, Rsite2 predicted 7 putative functional sites for the Diels-Alder ribozyme. We successfully predicted all of the 3 known functional sites (Table 2; Fig. 5). The result shows that Rsite2 has a sensitivity of 100% (3/3) and a PPV of 43% (3/7).

### Predicting the functional sites of the *S. cerevisiae* RNase P

**Figure 6:**
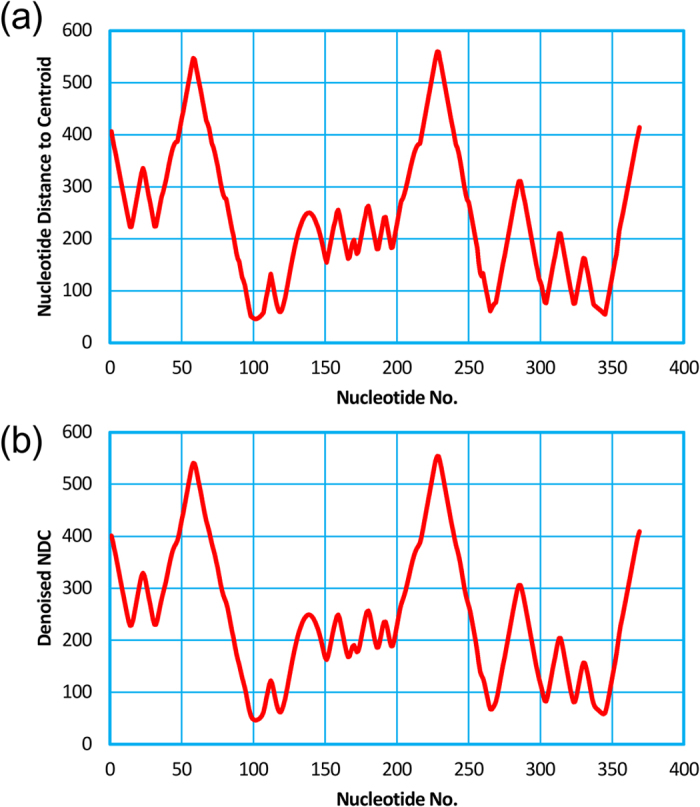
The raw nucleotide distance curve (**a**) and the smoothed nucleotide distance curve (**b**) of the *S. cerevisiae* RNase P.

**Figure 7:**
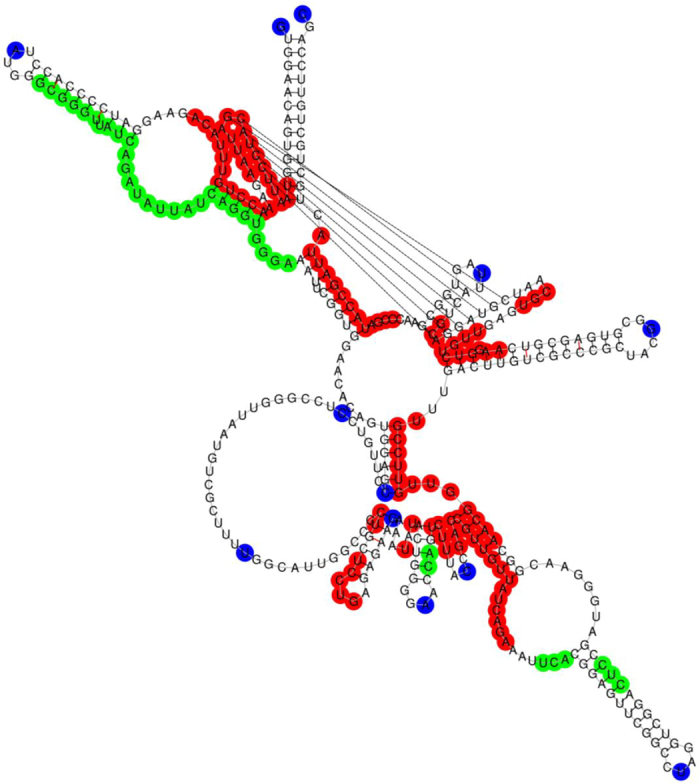
The predicted functional sites of the *S. cerevisiae* RNase P in a graphical representation. The figure shows the secondary structure of the *S. cerevisiae* RNase P. A predicted functional site is colored red on condition that it hits a known functional site, otherwise blue. And the missed known functional sites are in green color.

Moreover, we applied Rsite2 to RNAase P whose secondary structure and functional sites were experimentally identified. For doing so, we downloaded the secondary structure of RNase P from RNA STRAND^47^. And then we calculated the distance curve (Fig. 6). As a result, Rsite2 predicted 25 putative functional sites for the RNase P. Rsite2 successfully hit 13 of the 17 functional protein binding sites derived from RNase footprinting assays^48^ (Table 3; Fig. 7). It shows that Rsite2 achieves a sensitivity of 76% (13/17) and a PPV of 52% (13/25).

### Predicting the functional sites of human mitochondrial transcripts

Recently, by digital RNase footprinting, Liu *et al.*^49^ mapped the functional segments of human mitochondrial transcripts that participate in interactions with proteins. We wondered whether these regions can be predicted by Rsite2. For this purpose, we first retrieved the transcript sequences (Feb. 2009 GRCh37/hg19) on the mitochondrial chromosome (chrM) using the UCSC Table Browser data retrieval tool^50^ (http://genome.ucsc.edu/). RNAfold^51^ was then used to predict the secondary structures of these mitochondrial RNAs. Finally, Rsite2 predicted the putative functional sites for each of them. After careful comparison, we found that Rsite2 successfully hits 32 (74%) of the 43 functional segments in the mitochondrial RNAs from the UCSC database^50^. Due to the low ratio of footprint’s length to RNA’s length and the large number of predicted sites, and because in default Rsite2 merges sites no more than 2 nucleotides apart regardless of RNA’s length, PPVs range from 1% (1/72) to 44% (4/9) and the overall PPV is 7% (54/763) (Supplementary File 1).

## Discussion

It is increasingly needed to develop computational methods for the identification of ncRNA functional sites. We previously developed an ncRNA tertiary structure based method, Rsite. However it is largely limited because the tertiary structures of most ncRNAs are not available. In this study, we revealed that there is a significantly positive correlation between ncRNA secondary structures and tertiary structures. This observation gives clues that secondary structure-derived distance metrics could be used to predict their functional sites. Based on this observation, we then presented a secondary structure based computational method to predict candidate functional sites of ncRNAs. Since we are not able to assess Rsite2 systematically for the reason that there are not standard datasets that we can employ as benchmarks at the present stage, we showed the efficiency and accuracy of Rsite2 by applying it to three ncRNAs and a set of mitochondrial RNAs. Compared with the first version of Rsite, Rsite2 was improved in the following two aspects. Firstly, Rsite2 runs on the RNA secondary structures. It is known that RNA secondary structures are much easier and more feasible to be obtained by wet-lab or computational experiments than tertiary structures. This makes Rsite2 much more feasible and efficient. In addition, it would provide great convenience to the functional site prediction of large ncRNA, of which we just know about the primary sequence and the secondary structure, and facilitate the investigation of ncRNA functions. Secondly, Rsite2 provides the users with not only the source codes but also a web-server, which makes it very easy for users to predict the functional sites of their ncRNAs.

With simple single-stranded chains, ncRNAs have the ability to form certain common structural units which can serve as building blocks for subsequent elaborate construction of tertiary structures. So it makes sense that overall secondary structures of ncRNAs are positively correlated with their tertiary structures. However, due to the disparity between some predicted secondary structures and known secondary structures, their 2D-3D structure correlations are weak or even negative. For instance, an RNA pseudoknot with 3D domain swapping (PDB ID: 387D), whose length is 26, shows significantly negative correlation (Spearman rho = −0.82, p = 2e-07) if the secondary structure generated by RNAfold^51^ is used. Conversely, the correlation turns into a significantly positive one (Spearman rho = 0.69, p = 9e-05) once replaced with known 2D structure taken from RNA STRAND (http://www.rnasoft.ca/strand/)^47^.

Nonetheless, ncRNA secondary structure cannot perfectly substitute for tertiary structure in light of the inevitable information loss. It is quite difficult to precisely depict all of the more sophisticated tertiary structural motifs via secondary structural motifs. The more structural features there are unique to 3D-level, the more information loss comes from the replacement with 2D structure. Furthermore, we expect that taking other features such as sequence conservation and structure conservation into account would improve the current method.

From another perspective, the divergences between ncRNA secondary structure and tertiary structure might be hints for functional sites too. It is totally reasonable that nucleotides which seem apart in ncRNA secondary structure contact with each other in 3D space, forming a pocket that maybe catalyze chemical reactions, or a domain resembling other shapes, like a clamp. Besides, we hypothesize that, during the building process of tertiary structure, nucleotides folded inside out or outside in are candidates for functional sites. Further research about this is required.

The understanding of RNA folding principles is at an early stage^52^,^53^. When more details about RNA folding become available, we expect Rsite2 will be improved at that time. Finally, although limitations exist in the current method, we believe that the secondary structure based method (Rsite2) is an alternative solution for the prediction of the functional sites of ncRNAs.

## Methods

### The tertiary structure data of ncRNAs

To generally analyze the correlation between ncRNA secondary structure and tertiary structure, we first acquired the latest non-redundant PDB ID list of RNA-containing tertiary structures (Release 1.89, 2014-12-05) from NDB website (http://ndbserver.rutgers.edu/)^44^,^45^, and then downloaded from PDB server (http://www.rcsb.org/pdb/home/home.do)^19^ the corresponding biological assemblies of the equivalence class representatives. As a result, we obtained the tertiary structures of 1034 ncRNAs.

### Predicting the secondary structures of ncRNAs

From the above structure files of ncRNAs, we extracted the nucleotide sequences using an in-house python program. We then predicted the secondary structures of these ncRNAs using RNAfold^51^ (v1.8.5), an easy-to-use tool for RNA secondary structure prediction. It produced for each ncRNA sequence one PostScript file, from which we further fetched out the 2D coordinates of each ncRNA molecule.

### Calculating nucleotide location in ncRNA structures

We first obtained the 3D and the 2D coordinates from the PDB files and the PostScript files, respectively. We then calculated their molecular centroids based on the 3D and 2D coordinates. We presented two metrics to calculate the location of each nucleotide in an ncRNA. The first metric, nucleotide distance to centroid (NDC), is the Euclidean distances between any one nucleotide and the centroid. And we computed the sum of distances for each nucleotide between it and all the rest nucleotides in one RNA molecule as the second metric, called nucleotide distance sum (NDS). Both types of distances can quantitatively evaluate the location of one nucleotide. The outermost nucleotide has the biggest distance value and the innermost the smallest.

### Correlation analysis between the 2D and the 3D structures of ncRNAs

We performed Spearman correlation analysis for the 2D and the 3D nucleotide locations calculated using the above two metrics. Before the above process, we filtered out the ncRNAs with multiple RNA chains, those no longer than 20nt, those whose coordinates are incomplete, and those whose nucleotide composition does not cover all four kinds of bases.

### Identifying ncRNA functional sites

We used three ncRNAs (tRNA (Lys), Diels-Alder ribozyme and RNase P) and a set of human mitochondria transcripts with well-annotated functional sites to validate the accuracy of Rsite2. For a given ncRNA, Rsite2 first calculates the two metrics of nucleotide location defined above based on the 2D coordinates derived from the predicted secondary structure. Then, Rsite2 utilizes the algorithms of Rsite^40^ to predict functional sites. In Results, the distance metric is NDC in default.

### Algorithms of Rsite in brief

For an ncRNA with *n* nucleotides, we can get a distance curve (*D*) of length *n*, of which *D(i)* denotes the location of the *ith* nucleotide, and smooth it with a Gaussian filter. Then we identify the nucleotides that are the extreme points in the distance curve as the putative functional sites. And multiple points close (<= 2nt in default for their sequence positions) to each other are integrated into one functional site.

## Additional Information

**How to cite this article**: Zeng, P. and Cui, Q. Rsite2: an efficient computational method to predict the functional sites of noncoding RNAs. *Sci. Rep.* **6**, 19016; doi: 10.1038/srep19016 (2016).

## Supplementary Material

Supplementary Information

## Figures and Tables

**Table 1:** The known functional sites (FSs) and the Rsite2 hits of the tRNA (Lys).

Site No.	Known FS	Rsite2 hit	Description
**1**	1–4	1	Within acceptor stem(5’ end) Interacting with Ran Contacting Xpot Involved in recognition by RNase Z and RNase P Recognized by aminoacyl-tRNA synthetase
**2**	13–22	18	DHU loop Interacting with the mRNA-ribosome complex Contacting Xpot Involved in recognition by RNase P
**3**	34–36	35	Anticodon, Decoding mRNA codon Recognized by aminoacyl-tRNA synthetase Interacting with the mRNA-ribosome complex
**4**	49–51	49	Within TψC stem Binding site of elongation factor
**5**	53–61	58	TψC loop Interacting with the mRNA-ribosome complex Contacting Xpot Involved in recognition by RNase Z and RNase P Processed by a tRNA ψ55 pseudouridine synthase Affect 3’ end processing and tRNA structure
**6**	63–65	-	Within TψC stem Binding site of elongation factor Interacting with Ran Involved in recognition by RNase P
**7**	72–76	75,76	Aminoacylation site(3’ end) Recognized by aminoacyl-tRNA synthetase Interacting with the mRNA-ribosome complex Contacting Xpot Involved in recognition by RNase Z Processed by a CCA-adding enzyme

**Table 2:** The known functional sites (FSs) and the Rsite2 hits of the Diels-Alder ribozyme.

Site No.	Known FS	Rsite2 hit	Description
**1**	1–4	1	A part of the catalytic pocket
**2**	23–25	23	A part of the catalytic pocket
**3**	42–45	42	A part of the catalytic pocket

**Table 3:** The known functional sites (FSs) and the Rsite2 hits of the *S. cerevisiae* RNase P.

Site No.	Known FS	Rsite2 hit	Description
**1**	13–28,31–42,62–89	14,23,31	Interacting with Pop1/6/7
**2**	151–152,155–159,170,172, 183–184,186–189,194–209, 213–215,237–239,253–266, 298–307,311–313,323–325, 341–353	151,159,170,172, 186,196,266,304, 313,323,344	Interacting with Pop1

## References

[b1] Bertone P. *et al.* Global identification of human transcribed sequences with genome tiling arrays. Science 306, 2242–2246, doi: 10.1126/science.1103388 (2004). 15539566

[b2] Cheng J. *et al.* Transcriptional maps of 10 human chromosomes at 5-nucleotide resolution. Science 308, 1149–1154, doi: 10.1126/science.1108625 (2005). 15790807

[b3] Nagano T. & Fraser P. No-Nonsense Functions for Long Noncoding RNAs. Cell 145, 178–181, doi: 10.1016/j.cell.2011.03.014 (2011). 21496640

[b4] Lu M. *et al.* An analysis of human microRNA and disease associations. PloS One 3, e3420, doi: 10.1371/journal.pone.0003420 (2008). 18923704 PMC2559869

[b5] Li Y. *et al.* HMDD v2.0: a database for experimentally supported human microRNA and disease associations. Nucleic Acids Res 42, D1070–1074, doi: 10.1093/nar/gkt1023 (2014). 24194601 PMC3964961

[b6] Chen G. *et al.* LncRNADisease: a database for long-non-coding RNA-associated diseases. Nucleic Acids Res 41, D983–986, doi: 10.1093/nar/gks1099 (2013). 23175614 PMC3531173

[b7] Yang L., Froberg J. E. & Lee J. T. Long noncoding RNAs: fresh perspectives into the RNA world. Trends Biochem Sci 39, 35–43, doi: 10.1016/j.tibs.2013.10.002 (2014). 24290031 PMC3904784

[b8] Mo Y. Y. MicroRNA regulatory networks and human disease. Cell Mol Life Sci 69, 3529–3531, doi: 10.1007/s00018-012-1123-1 (2012). 22926413 PMC3475759

[b9] Lee J. T. Epigenetic regulation by long noncoding RNAs. Science 338, 1435–1439, doi: 10.1126/science.1231776 (2012). 23239728

[b10] Li Y. & Zhang Z. Computational Biology in microRNA. Wires RNA 6, 435–452, doi: 10.1002/wrna.1286 (2015). 25914300

[b11] Mercer T. R. & Mattick J. S. Structure and function of long noncoding RNAs in epigenetic regulation. Nat Struct Mol Biol 20, 300–307, doi: 10.1038/nsmb.2480. (2013). 23463315

[b12] Mortimer S. A., Kidwell M. A. & Doudna J. A. Insights into RNA structure and function from genome-wide studies. Nat Rev Genet 15, 469–479, doi: 10.1038/nrg3681 (2014). 24821474

[b13] Jones C. P. & Ferre-D’Ama A. R. RNA quaternary structure and global symmetry. Trends Biochem Sci 40, 211–220, doi: 10.1016/j.tibs.2015.02.004 (2015). 25778613 PMC4380790

[b14] Ge P. & Zhang S. Computational analysis of RNA structures with chemical probing data. Methods, doi: 10.1016/j.ymeth.2015.02.003 (2015). PMC4437859 25687190

[b15] Siegfried N. A., Busan S., Rice G. M., Nelson J. A. & Weeks K. M. RNA motif discovery by SHAPE and mutational profiling (SHAPE-MaP). Nat Methods 11, 959–965, doi: 10.1038/nmeth.3029 (2014). 25028896 PMC4259394

[b16] Nilsen T. W. RNase footprinting to map sites of RNA-protein interactions. Cold Spring Harbor protocols 2014, 677–682, doi: 10.1101/pdb.prot080788 (2014). 24890210

[b17] Tullius T. D. & Greenbaum J. A. Mapping nucleic acid structure by hydroxyl radical cleavage. Curr Opin Chem Biol 9, 127–134, doi: 10.1016/j.cbpa.2005.02.009 (2005). 15811796

[b18] Juzumiene D., Shapkina T., Kirillov S. & Wollenzien P. Short-Range RNA-RNA Crosslinking Methods to Determine rRNA Structure and Interactions. Methods 25, 333–343 (2001). 11860287 10.1006/meth.2001.1245

[b19] Berman H. M. *et al.* The Protein Data Bank. Nucleic Acids Res 28, 235–242 (2000). 10592235 10.1093/nar/28.1.235 PMC102472

[b20] Hermjakob H. *et al.* IntAct: an open source molecular interaction database. Nucleic Acids Res 32, D452–455, doi: 10.1093/nar/gkh052 (2004). 14681455 PMC308786

[b21] Bader G. D., Betel D. & Hogue C. W. BIND: the Biomolecular Interaction Network Database. Nucleic Acids Res 31, 248–250 (2003). 12519993 10.1093/nar/gkg056 PMC165503

[b22] Xenarios I. *et al.* DIP: the database of interacting proteins. Nucleic Acids Res 28, 289–291 (2000). 10592249 10.1093/nar/28.1.289 PMC102387

[b23] Finn R. D. *et al.* The Pfam protein families database. Nucleic Acids Res 38, D211–222, doi: 10.1093/nar/gkp985 (2010). 19920124 PMC2808889

[b24] Sigrist C. J. *et al.* PROSITE, a protein domain database for functional characterization and annotation. Nucleic Acids Res 38, D161–166, doi: 10.1093/nar/gkp885 (2010). 19858104 PMC2808866

[b25] Ivanisenko V. A., Pintus S. S., Grigorovich D. A. & Kolchanov N. A. PDBSite: a database of the 3D structure of protein functional sites. Nucleic Acids Res 33, D183–187, doi: 10.1093/nar/gki105 (2005). 15608173 PMC540059

[b26] Porter C. T., Bartlett G. J. & Thornton J. M. The Catalytic Site Atlas: a resource of catalytic sites and residues identified in enzymes using structural data. Nucleic Acids Res 32, D129–D133, doi: 10.1093/nar/gkh028 (2004). 14681376 PMC308762

[b27] Dukka B. K. Structure-based Methods for Computational Protein Functional Site Prediction. Comput Struct Biotechnol J 8, e201308005, doi: 10.5936/csbj.201308005 (2013). 24688745 PMC3962076

[b28] Somarowthu S. & Ondrechen M. J. POOL server: machine learning application for functional site prediction in proteins. Bioinformatics 28, 2078–2079, doi: 10.1093/bioinformatics/bts321 (2012). 22661648 PMC3400966

[b29] Yang Z. R., Wang L., Young N., Trudgian D. & Chou K. C. Pattern recognition methods for protein functional site prediction. Curr Protein Pept Sc 6, 479–491 (2005). 16248799 10.2174/138920305774329322

[b30] La D., Sutch B. & Livesay D. R. Predicting protein functional sites with phylogenetic motifs. Proteins 58, 309–320, doi: 10.1002/prot.20321 (2005). 15573397

[b31] La D. & Livesay D. R. MINER: software for phylogenetic motif identification. Nucleic Acids Res 33, W267–270, doi: 10.1093/nar/gki465 (2005). 15980467 PMC1160226

[b32] de Castro E. *et al.* ScanProsite: detection of PROSITE signature matches and ProRule-associated functional and structural residues in proteins. Nucleic Acids Res 34, W362–365, doi: 10.1093/nar/gkl124 (2006). 16845026 PMC1538847

[b33] Amitai G. *et al.* Network analysis of protein structures identifies functional residues. J Mol Biol 344, 1135–1146, doi: 10.1016/j.jmb.2004.10.055 (2004). 15544817

[b34] Verspoor K. M., Cohn J. D., Ravikumar K. E. & Wall M. E. Text mining improves prediction of protein functional sites. PloS One 7, e32171, doi: 10.1371/journal.pone.0032171 (2012). 22393388 PMC3290545

[b35] Kinoshita K. & Nakamura H. Identification of the ligand binding sites on the molecular surface of proteins. Protein Sci 14, 711–718, doi: 10.1110/ps.041080105 (2005). 15689509 PMC2279290

[b36] Greaves R. & Warwicker J. Active site identification through geometry-based and sequence profile-based calculations: burial of catalytic clefts. J Mol Bio 349, 547–557, doi: 10.1016/j.jmb.2005.04.018 (2005). 15882869

[b37] del Sol A., Fujihashi H., Amoros D. & Nussinov R. Residue centrality, functionally important residues, and active site shape: analysis of enzyme and non-enzyme families. Protein Sci 15, 2120–2128, doi: 10.1110/ps.062249106 (2006). 16882992 PMC2242611

[b38] Chea E. & Livesay D. R. How accurate and statistically robust are catalytic site predictions based on closeness centrality? BMC Bioinformatics 8, 153, doi: 10.1186/1471-2105-8-153 (2007). 17498304 PMC1876251

[b39] Diederichs S. The four dimensions of noncoding RNA conservation. Trends Genet 30, 121–123, doi: 10.1016/j.tig.2014.01.004 (2014). 24613441

[b40] Zeng P., Li J., Ma W. & Cui Q. Rsite: a computational method to identify the functional sites of noncoding RNAs. Scientific Rep 5, 9179, doi: 10.1038/srep09179 (2015). PMC4361870 25776805

[b41] Das R. & Baker D. Automated *de novo* prediction of native-like RNA tertiary structures. P Natl Acad Sci USA 104, 14664–14669, doi: 10.1073/pnas.0703836104 (2007). PMC1955458 17726102

[b42] Frellsen J. *et al.* A probabilistic model of RNA conformational space. PLoS Comput Biol 5, e1000406, doi: 10.1371/journal.pcbi.1000406 (2009). 19543381 PMC2691987

[b43] Sharma S., Ding F. & Dokholyan N. V. iFoldRNA: three-dimensional RNA structure prediction and folding. Bioinformatics 24, 1951–1952, doi: 10.1093/bioinformatics/btn328 (2008). 18579566 PMC2559968

[b44] Leontis N. & Zirbel C. In RNA 3D Structure Analysis and Prediction Vol. 27 Nucleic Acids and Molecular Biology (eds Leontis Neocles & Westhof Eric) Ch. 13, 281–298 (Springer Berlin: Heidelberg, , 2012).

[b45] Coimbatore Narayanan B. *et al.* The Nucleic Acid Database: new features and capabilities. Nucleic Acids Res 42, D114–122, doi: 10.1093/nar/gkt980 (2014). 24185695 PMC3964972

[b46] Dabney A., Storey J. D. & Warnes G. Q-value estimation for false discovery rate control. Medicine 344, 539–548 (2004).

[b47] Andronescu M., Bereg V., Hoos H. & Condon A. RNA STRAND: The RNA Secondary Structure and Statistical Analysis Database. BMC Bioinformatics 9, 340 (2008). 18700982 10.1186/1471-2105-9-340 PMC2536673

[b48] Fagerlund R. D., Perederina A., Berezin I. & Krasilnikov A. S. Footprinting analysis of interactions between the largest eukaryotic RNase P/MRP protein Pop1 and RNase P/MRP RNA components. RNA 21, 1591–1605, doi: 10.1261/rna.049007 (2015). 26135751 PMC4536320

[b49] Liu G. *et al.* Mapping of mitochondrial RNA-protein interactions by digital RNase footprinting. Cell Rep 5, 839–848 (2013). 24183674 10.1016/j.celrep.2013.09.036

[b50] Karolchik D. *et al.* The UCSC Table Browser data retrieval tool. Nucleic Acids Res 32, D493–D496 (2004). 14681465 10.1093/nar/gkh103 PMC308837

[b51] Hofacker I. L. *et al.* Fast folding and comparison of RNA secondary structures. Monatsh Chem 125, 167–188, doi: 10.1007/BF00818163 (1994).

[b52] Holbrook S. R. Structural principles from large RNAs. Annu Rev Biophys 37, 445–464, doi: 10.1146/annurev.biophys.36.040306.132755 (2008). 18573090

[b53] Zwieb C. The principles of RNA structure architecture. Methods Mol Biol 1097, 33–43, doi: 10.1007/978-1-62703-709-9_2 (2014). 24639153

