# Supplementary material for: Rsite2: an efficient computational method to predict the functional sites of noncoding RNAs
Source: Sci Rep. 2016 Jan 11;6:19016. doi: 10.1038/srep19016 (PMC4707467; doi:10.1038/srep19016)
Supplement: Supplementary Information [file srep19016-s1.doc]

**Rsite2: an efficient computational method to predict the functional sites of**

**noncoding RNAs**

Pan Zeng and Qinghua Cui^*^

Department of Biomedical Informatics, Centre for Noncoding RNA Medicine, School of Basic Medical Sciences, Peking University, 38 Xueyuan Rd, Beijing, 100191, China

* To whom the correspondence should be addressed:

[cuiqinghua@bjmu.edu.cn](mailto:cuiqinghua@bjmu.edu.cn)

Supplementary File 1. The known functional sites (FSs) and the Rsite2 hits of human mitochondrial transcripts.

Ensembl Transcript ID Chromosome Transcript Start Transcript End Strand Footprint Start Footprint End Strand Relative Footprint Start Relative Footprint End Rsite2 Predicted Site

Ensembl Transcript ID Chromosome Transcript Start Transcript End Transcript Length Strand Footprint Start Footprint End Footprint Length Strand Relative Footprint Start Relative Footprint End Rsite2 Hit PPV(%) Footprint Length Ratio

ENST00000389680 chrM 646 1599 954 + 1054 1085 32 + 409 440 440 1.39% 3.35%

ENST00000386347 chrM 3229 3303 75 + 3298 3310 13 + 70 82 75 20.00% 17.33%

ENST00000361390 chrM 3306 4261 956 + 3437 3445 9 + 132 140 - 10.00% 0.94%

ENST00000361390 chrM 3306 4261 956 + 3854 3866 13 + 549 561 - 10.00% 1.36%

ENST00000361390 chrM 3306 4261 956 + 3904 3912 9 + 599 607 602 10.00% 0.94%

ENST00000361390 chrM 3306 4261 956 + 4125 4133 9 + 820 828 822 10.00% 0.94%

ENST00000361390 chrM 3306 4261 956 + 4153 4177 25 + 848 872 849;850;857;864 10.00% 2.62%

ENST00000361390 chrM 3306 4261 956 + 4204 4214 11 + 899 909 901;902 10.00% 1.15%

ENST00000387365 chrM 4262 4330 69 + 4292 4300 9 + 31 39 34 11.11% 13.04%

ENST00000387377 chrM 4401 4468 68 + 4395 4419 25 + -5 19 11;19 22.22% 36.76%

ENST00000361453 chrM 4469 5510 1042 + 4628 4636 9 + 160 168 - 5.33% 0.86%

ENST00000361453 chrM 4469 5510 1042 + 4927 4935 9 + 459 467 460;461;463;467 5.33% 0.86%

ENST00000361453 chrM 4469 5510 1042 + 4984 4993 10 + 516 525 522;523 5.33% 0.96%

ENST00000361453 chrM 4469 5510 1042 + 5019 5031 13 + 551 563 - 5.33% 1.25%

ENST00000361453 chrM 4469 5510 1042 + 5309 5319 11 + 841 851 849 5.33% 1.06%

ENST00000361453 chrM 4469 5510 1042 + 5347 5355 9 + 879 887 - 5.33% 0.86%

ENST00000361624 chrM 5903 7444 1542 + 5965 5973 9 + 63 71 65;67 2.78% 0.58%

ENST00000361624 chrM 5903 7444 1542 + 6770 6788 19 + 868 886 879;883 2.78% 1.23%

ENST00000361624 chrM 5903 7444 1542 + 7427 7446 20 + 1525 1544 - 2.78% 1.30%

ENST00000361739 chrM 7585 8268 684 + 7831 7845 15 + 247 261 249;257 3.85% 2.19%

ENST00000387421 chrM 8294 8363 70 + 8343 8364 22 + 50 71 51;57;63;70 44.44% 31.43%

ENST00000361851 chrM 8365 8571 207 + 8520 8528 9 + 156 164 159 25.00% 4.35%

ENST00000361851 chrM 8365 8571 207 + 8538 8552 15 + 174 188 176 25.00% 7.25%

ENST00000361899 chrM 8526 9206 681 + 8520 8528 9 + -5 3 2 13.51% 1.32%

ENST00000361899 chrM 8526 9206 681 + 8538 8552 15 + 13 27 14 13.51% 2.20%

ENST00000361899 chrM 8526 9206 681 + 8667 8701 35 + 142 176 143;163 13.51% 5.14%

ENST00000361899 chrM 8526 9206 681 + 8844 8856 13 + 319 331 - 13.51% 1.91%

ENST00000361899 chrM 8526 9206 681 + 8861 8873 13 + 336 348 - 13.51% 1.91%

ENST00000361899 chrM 8526 9206 681 + 9091 9104 14 + 566 579 579 13.51% 2.06%

ENST00000362079 chrM 9206 9989 784 + 9933 9942 10 + 728 737 733;735 1.45% 1.28%

ENST00000387429 chrM 9990 10057 68 + 9997 10017 21 + 8 28 12;18;26 42.86% 30.88%

ENST00000361227 chrM 10058 10403 346 + 10064 10089 26 + 7 32 - 7.14% 7.51%

ENST00000361227 chrM 10058 10403 346 + 10257 10267 11 + 200 210 200 7.14% 3.18%

ENST00000361381 chrM 10759 12136 1378 + 10797 10825 29 + 39 67 40;44 7.61% 2.10%

ENST00000361381 chrM 10759 12136 1378 + 10910 10920 11 + 152 162 159 7.61% 0.80%

ENST00000361381 chrM 10759 12136 1378 + 10981 11017 37 + 223 259 244;251;257;258 7.61% 2.69%

ENST00000361381 chrM 10759 12136 1378 + 12067 12083 17 + 1309 1325 1309 7.61% 1.23%

ENST00000387441 chrM 12137 12205 69 + 12180 12207 28 + 44 71 53;65;69 42.86% 40.58%

ENST00000361789 chrM 14746 15886 1141 + 14823 14831 9 + 78 86 - 4.35% 0.79%

ENST00000361789 chrM 14746 15886 1141 + 14877 14905 29 + 132 160 139;141;143;149;155;158 4.35% 2.54%

ENST00000361789 chrM 14746 15886 1141 + 15232 15240 9 + 487 495 - 4.35% 0.79%

ENST00000387460 chrM 15887 15952 66 + 15936 15953 18 + 50 67 58;61;66 33.33% 27.27%

ENST00000361681 chrM 14148 14672 525 - 14340 14354 15 - 193 207 207 3.45% 2.86%
